# Hackathons as a means of accelerating scientific discoveries and knowledge transfer

**DOI:** 10.1101/gr.228460.117

**Published:** 2018-05

**Authors:** Amel Ghouila, Geoffrey Henry Siwo, Jean-Baka Domelevo Entfellner, Sumir Panji, Katrina A. Button-Simons, Sage Zenon Davis, Faisal M. Fadlelmola, The DREAM of Malaria Hackathon Participants, Michael T. Ferdig, Nicola Mulder

**Affiliations:** 1 Institut Pasteur de Tunis, LR11IPT02, Laboratory of Transmission, Control and Immunobiology of Infections (LTCII), 1002 Tunis-Belvédère, Tunisia;; 2 IBM Research Africa, 2001, Johannesburg, South Africa;; 3 Center for Research Computing, University of Notre Dame, Notre Dame, Indiana 46556, USA;; 4 South African National Bioinformatics Institute/Medical Research Council of South Africa Bioinformatics Unit, University of the Western Cape, Bellville 7535, Cape Town, South Africa;; 5 Computer Science Department, University of the Western Cape, Bellville 7535, Cape Town, South Africa;; 6 Computational Biology Division, Department of Integrative Biomedical Sciences, Institute of Infectious Disease and Molecular Medicine, University of Cape Town, 7925, Cape Town, South Africa;; 7 Eck Institute for Global Health, University of Notre Dame, Notre Dame, Indiana 46556, USA;; 8 Centre for Bioinformatics and Systems Biology, Faculty of Science, University of Khartoum, Khartoum 321, and Future University of Sudan, Khartoum, 10553, Sudan; 12 System and Data Engineering Team Abdelmalek Essaadi University, ENSA, 90000 Tangier, Morocco and University of Liverpool, Liverpool, Liverpool L69 7ZB, UK; 13 Noguchi Memorial Institute for Medical Research College of Health Sciences, University of Ghana, LG 581 Legon, Ghana; 14 Laboratory of Bioinformatics, Biomathematics and Biostatistics (LR16IPT09), Institut Pasteur of Tunis, University of Tunis El Manar, 1002 Tunis, Tunisia; 15 IBM Research Africa, 2001 Johannesburg, South Africa; 16 Department of Computer & Information Sciences & Nigeria Covenant University Bioinformatics Research (CUBRe) Covenant University, 112233 Ota, Ogun State, Nigeria; 17 Faculty of Agriculture, Zagazig University, 44519 Sharkia, Egypt; 18 System and Data Engineering Team Abdelmalek Essaadi University, ENSA, 90000 Tangier, Morocco; 19 Group of Biostatistics, Bioinformatics and Modeling (G4-BBM), Institut Pasteur de Dakar, 12500 Dakar, Senegal; 20 Malawi-Liverpool-Wellcome Trust Clinical Research Programme, Queen Elizabeth Central Hospital, Chichiri Blantyre 3, Malawi; 21 IBM Research Africa, Karen 00200 Nairobi, Kenya; 22 As members of the H3ABioNet Consortium's Research Working Group, and members of the H3Africa Consortium

## Abstract

Scientific research plays a key role in the advancement of human knowledge and pursuit of solutions to important societal challenges. Typically, research occurs within specific institutions where data are generated and subsequently analyzed. Although collaborative science bringing together multiple institutions is now common, in such collaborations the analytical processing of the data is often performed by individual researchers within the team, with only limited internal oversight and critical analysis of the workflow prior to publication. Here, we show how hackathons can be a means of enhancing collaborative science by enabling peer review before results of analyses are published by cross-validating the design of studies or underlying data sets and by driving reproducibility of scientific analyses. Traditionally, in data analysis processes, data generators and bioinformaticians are divided and do not collaborate on analyzing the data. Hackathons are a good strategy to build bridges over the traditional divide and are potentially a great agile extension to the more structured collaborations between multiple investigators and institutions.

In September 2016, a group of 23 researchers and PhD students from around Africa gathered for a 1-wk hackathon aimed at developing an open innovation framework to engage young scientists across Africa. The goal of the hackathon was threefold: (1) assess the possibility of utilizing transcriptional data sets of several isolates of the human malaria parasite, *Plasmodium falciparum*, for a future open innovation challenge on predicting mechanisms of artemisinin resistance; (2) engage young scientists in Africa in gaining skills on the utility of genomic approaches in the prediction of drug resistance (a looming threat to the eradication of infectious diseases, including malaria); and (3) enable African scientists to gain new skills and experience by working in multidisciplinary and multicultural teams. To facilitate the hackathon, three organizations from different sectors with diverse expertise joined forces: the H3Africa Bioinformatics Network (H3ABioNet), the University of Notre Dame, and IBM Research Africa. H3ABioNet (Mulder et al. 2016) is a Pan-African bioinformatics network composed of 32 bioinformatics research groups distributed among 14 African countries and forming part of the larger H3Africa initiative (The H3Africa Consortium et al. 2014), IBM Research Africa is one of the first private industrial research organizations in Africa, and the University of Notre Dame is an academic and research university in the United States.

The University of Notre Dame is leading an effort to support a Dialogue on Reverse-Engineering Assessment and Methods (DREAM) (Stolovitzky et al. 2007) challenge on the prediction of artemisinin sensitivity of malaria parasite isolates using transcriptional data sets. The DREAM challenges are a series of annual crowdsourcing challenges during which biological data sets are released to the international community to build computational models that address specific biological questions (Stolovitzky et al. 2007). In contrast to typical crowdsourcing challenges, the University of Notre Dame, IBM Research Africa, and H3ABioNet collaborated and gathered researchers with different levels of expertise to assess the idea of opening up research data sets prior to publication for use in a data mining workshop. Such a forum, which we refer to as the DREAM of Malaria Hackathon, provides a number of benefits over the conventional research process: (1) opening up research data prior to publication to independent researchers provides an opportunity for unbiased assessment and critique of study design and data quality; (2) unpublished research data coupled with specific scientific questions can be used to train young scientists on hypothesis generation and technical skills for real-world data analysis problems; and (3) unpublished research data, when used in such a setting, exposes young scientists to the possibility of participating in scientific discoveries. The latter is especially important in Africa, where local researchers are often engaged in data collection with the intellectual aspects of many research projects, such as data analysis, being performed overseas. In contrast, this hackathon presented one of the rare reversals in which data from outside Africa was first analyzed in Africa and not the other way around (Fig. 1). Although the approach may not always be applicable in many cases of research, we hope that such collaborative exchanges become more common, especially in big data projects in which it could help reduce bias, enable reproducibility of research results, and engage emerging African scientists in tackling pertinent scientific challenges while gaining technical skills working in a multidisciplinary environment. Working in collaborative, multidisciplinary teams is relatively novel in Africa, where scientists are often isolated and tend to collaborate more with scientists abroad. The DREAM of Malaria Hackathon, unlike the other hackathons such as the Sana Project (Angelidis et al. 2016), is a hypothesis-driven research hackathon in which a specific scientific question is posed to the participants. Unlike Sana, it is geared toward improving basic science understanding in a specific topic and skills development.

**Figure 1.**
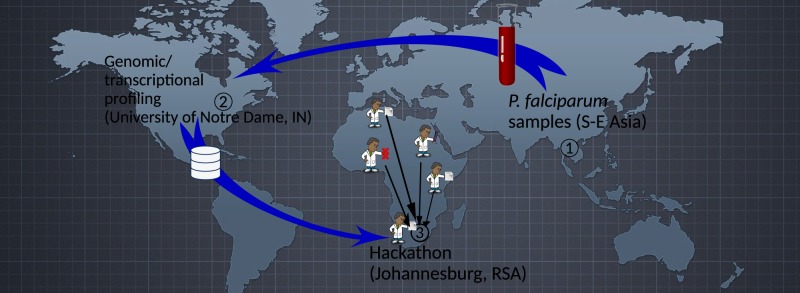
The DREAM of Malaria Hackathon as one of the phases of an international research effort spanning three continents. *Plasmodium falciparum* samples originated from Southeast Asia, data generators were at the University of Notre Dame (USA), and all other participants traveled from various African countries to the hackathon venue at IBM Research Africa, Johannesburg, South Africa.

## Results and discussion

### Multidisciplinary teams enhanced the scientific reflections and the exploration of novel ideas

The teams undertook different strategies toward tackling the challenge that was presented.

One team came up with distinct ways of modeling the relationship between gene expression patterns of the samples and their artemisinin sensitivity using a range of statistical and machine learning methods, including linear regression and support vector machines (SVM). The teams also proposed new methods for modeling gene expression data. Although these methods are the subject of a publication that is currently under preparation, we provide examples here because they led to dialogue between team members and will require further validation and peer review.

One of the new approaches included a method for estimating the developmental stage of a sample and using the estimated stage as a variable in machine learning models for predicting drug sensitivity from gene expression. This method could help address one of the challenges with the analysis of gene expression data from cell cultures, because often cultures are asynchronous, which could lead to differential gene expression associated with developmental progression rather than conditions of interest. Another new method developed by a team member leveraged gene expression data from human cancer cell lines perturbed with artemisinin to identify genes in the malaria parasite that are homologous to the human genes that respond or bind to artemisinin. The team member also went on to develop a new method that leveraged the virtual docking of artemisinin to proteins with 3D structures and identified homologous proteins in *P. falciparum* that bound the drug in the simulations. These innovative approaches stemming from group interactions demonstrate the potential for hackathons to accelerate out-of-the-box ideas created by multidisciplinary teams in a short time. The source code developed during the hackathon is publicly available on Synapse (https://www.synapse.org/Portal.html#!Synapse:syn11691127).

### Scientific outcomes of the hackathon

**Figure 2.**
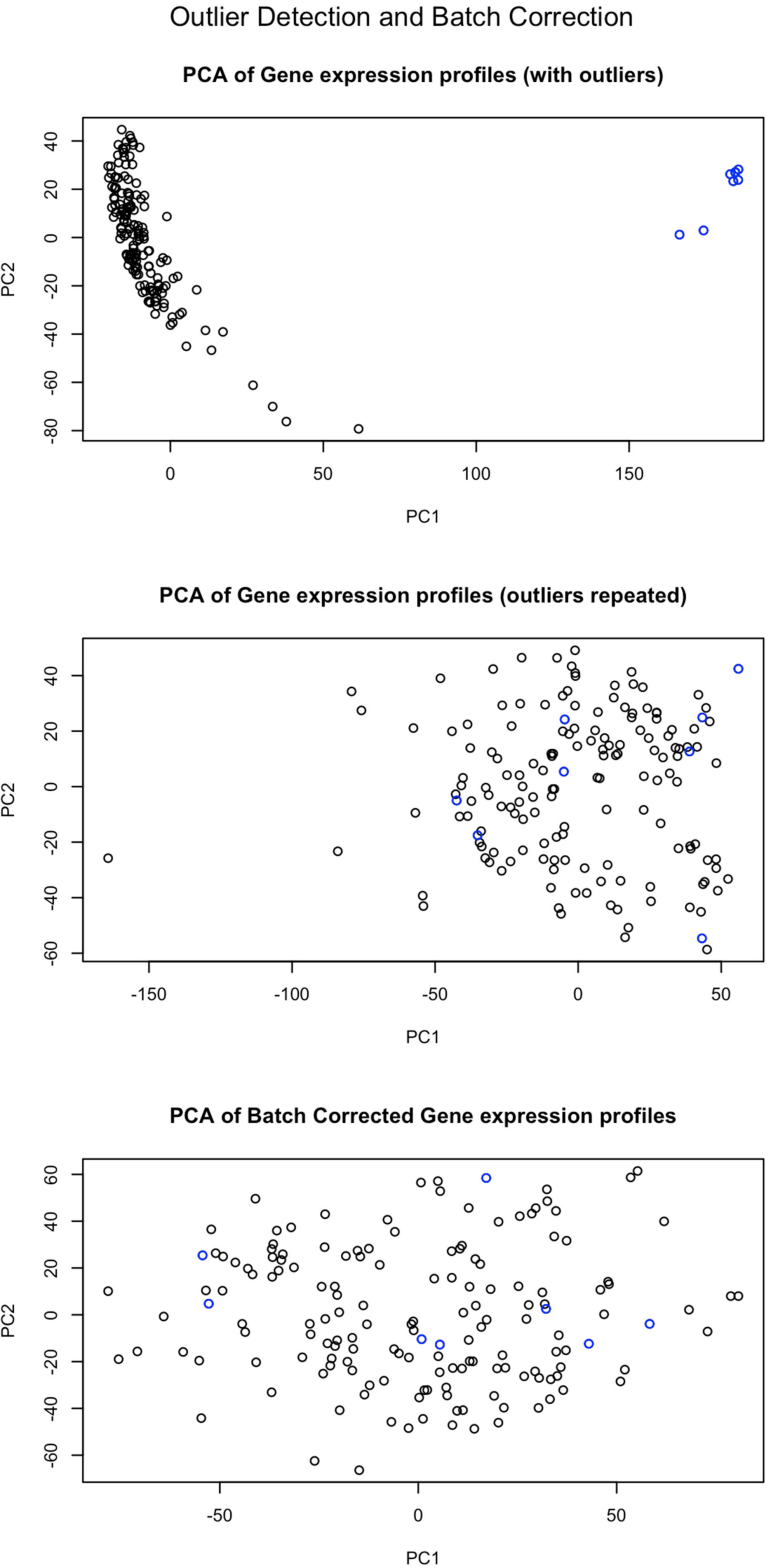
PCA plots of the expression data with the outliers shown in blue. The *top* plot shows the separate clustering of these samples, and the other plots show how outliers have been removed and that the repeated experiments have improved quality.

During the hackathon, participants started by focusing on cleaning up and quality-checking the data based on the raw data provided. This step allowed the different teams to detect some discrepancies in the data. Through rigorous analysis, the teams discovered that a subsample of the experiments were outliers in a principal component analysis (PCA) of all the gene expression data as illustrated in Figure 2. They showed that all experiments derived from one of the arrays were outliers and were therefore potentially artifacts. After long discussions with the data generators, who initially used the preanalysis provided by Agilent software, this observation led to a recommendation to the data generators to repeat the experiments on new arrays after the hackathon. As a result, the data generators performed new experiments after the hackathon, which were then reevaluated by the teams and this time shown to be of high quality. Figure 2 shows that the identified outliers were successfully removed in the newly performed experiment.

Data generators do not usually have easy access to this kind of feedback about the quality of the data. One of the most relevant strengths of this kind of hackathon is enabling a group of researchers with different background skills to work and reflect on the data quality to ensure that high-quality data is provided to the community. Bringing together data generators and analysts to spend time working on data is uncommon, and it allowed each group to learn more about the other group's domain and contribute to building bridges between them. This approach could be adopted for all the data sets that are opened for data mining challenges to enhance the quality of the analyses. Moreover, this collaborative data preprocessing and quality analysis approach could influence the future data generation process by offering the data generators new guidelines to be taken into consideration at early stages of their research.

Another team did a genome-wide comparison of all isolates, which revealed that significant differences existed between isolates and that within some of the isolates, the signatures of the two biological replicates did not always correlate. Moreover, the same team discovered that major differences between the staging values obtained for different isolates corresponding to the same developmental time point suggested that rather than analyzing samples at 6- and 24-h time points separately, an alternative approach of analyzing them together while adding two covariates corresponding to the staging information, could lead to better prediction models.

After these quality-control and early analysis steps, participants designed regression models and used them to predict the IC_50_ values from the different covariates (gene expression values, staging information, and some metadata). Several automatic feature selection algorithms were tested by the different teams: partial least squares, ridge regression, regularized regression, random forests, and LASSO. Some predictive models gave encouraging results. Overall, the results obtained showed that extracting a signal from this data set to predict the efficiency of dihydroartemisinin (DHA) on an isolate given its transcriptomic response is nontrivial, but not impossible. Nevertheless, the work carried out by the three teams during and after the hackathon paved the way for further investigation by ensuring robust quality control of the data set as well as providing the community with some predictive models. It gave baseline predictive performance values that will serve as a threshold to assess and filter the results that will be generated by the participants of the DREAM challenge.

### Data analysis hackathon as a cross-domain, peer-learning environment

Hackathons have emerged recently as a new way of creating products, advancing health care, and promoting rapid innovation (Groen and Calderhead 2015). The concept has grown since its initiation and is used by industries, scientists, and others to solve a wide variety of problems and to develop new strategies within a short period of time. The new scientific hackathon format undertaken here, which focuses on analyzing a specific data set, is a good strategy to obtain a deep analysis of the data and promotes learning based on problem solving. The participants have the opportunity to put their knowledge into practice by working in a collaborative environment. Moreover, working in multidisciplinary teams allows for combination of skills from researchers who might not have had the chance to work closely together to build solutions through integrating small contributions from participants with different backgrounds. For the majority of the participants, this was the first time they had ever taken part in a hackathon, and it highlighted to them the importance of multidisciplinary collaborations.

From an educational point of view, knowledge transfer is an important part of these data analysis–based hackathons enabled by working with peers across domains (Lapp et al. 2007; Groen and Calderhead 2015; Nandi and Mandernach 2016). It offers participants a new and interesting education and learning environment, especially for PhD students and early career researchers, by providing the opportunity to learn how to contribute to collaborative scientific projects.

**Figure 3.**
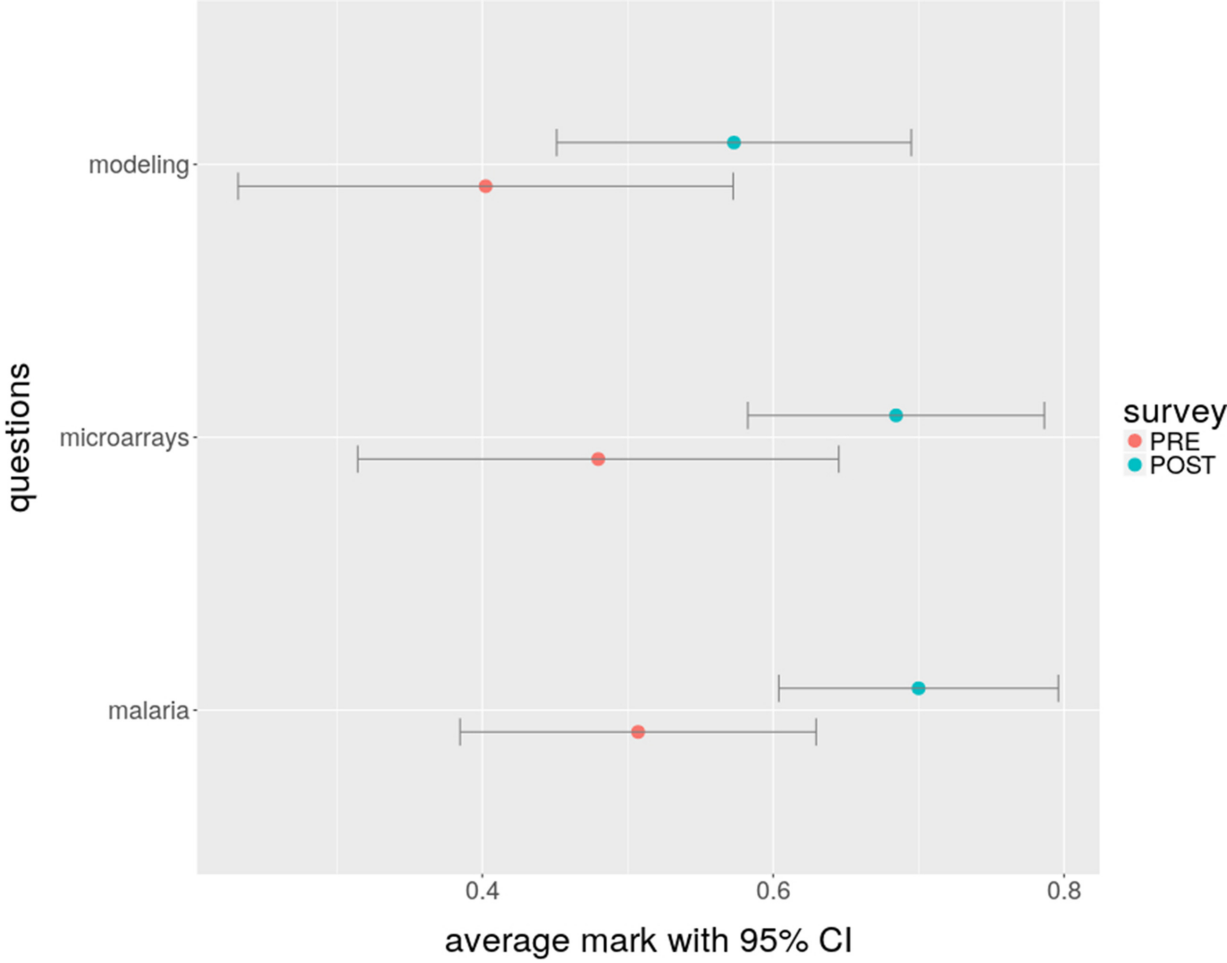
Average normalized survey marks with confidence intervals for the three broad categories of questions, at the beginning (survey “PRE”) and at the end (survey “POST”) of the hackathon.

**Figure 4.**
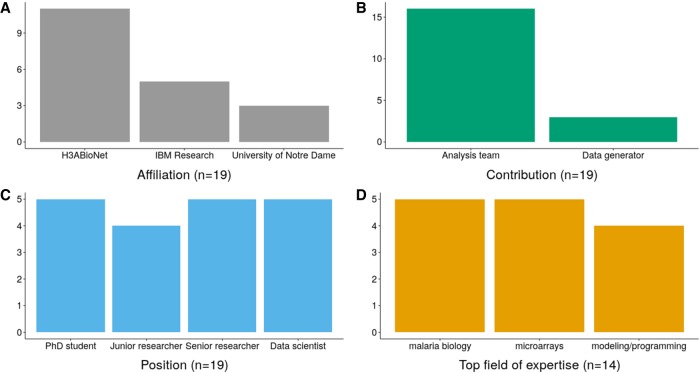
Breakdown of the 19 participants of the hackathon: (*A*) affiliation of the participants; (*B*) contribution to the hackathon; (*C*) type of position in their home institution; and (*D*) top field of expertise (data here are only for the 14 participants that filled both entry and exit surveys).

In order to assess knowledge transfer outcomes at the hackathon, all participants were asked to fill in a survey focusing on the various skills required for the data analysis during the hackathon. The major types of questions posed to participants fell into three different topics: microarray analysis, the biology of malaria and drug resistance, and data analysis with modeling algorithms. The same survey was administered to the participants at the end of the hackathon (for the survey questions, see Supplemental File S1). Given the small set of participants in the hackathon, testing for the statistical significance of the knowledge transfer is not possible. Nevertheless, the increase in knowledge of the participants, as illustrated by the results of the short survey that was conducted (Fig. 3), clearly supports the idea of knowledge transfer between participants. Fourteen participants completed both surveys, with a breakdown according to their top field of expertise shown in Figure 4.

### Challenges of the hackathon framework

Although the participants found that it was impressively easy to work with scientists from different backgrounds from the first day of the hackathon, there were a number of challenges. For example, online discussions before and after the hackathon were difficult to coordinate due to different time zones and schedules for participants, and technical challenges related to internet connectivity. It has been more difficult for participants to work together after their return to their respective laboratories or universities. In the future, it will be important to build some form of interpersonal “glue” within each team during the hackathon to maximize chances that people are going to continue to work together after the hackathon. Alternatively, the hackathon could be longer so that the required follow-up work is minimal. Despite these challenges, some post-hackathon activities have continued, albeit at a slower pace. Although not every hackathon participant is still actively contributing, the findings led the data generators to obtain new data that will be used in the open innovation challenge, and the hackathon outcomes are evolving continuously and influencing the organization of the challenge. Some of the findings were presented at the Sage Assembly and DREAM RECOMB meeting in 2017.

Funding for hackathons involving participants from various countries puts limitations on when and how often such hackathons may be organized. Fortunately, in the case of this hackathon, funding was available through H3ABioNet to support travel and accommodation of all participants, and IBM Research Africa provided a meeting venue and collaboration spaces.

## Methods

### Timeline of DREAM of Malaria Hackathon

**Figure 5.**
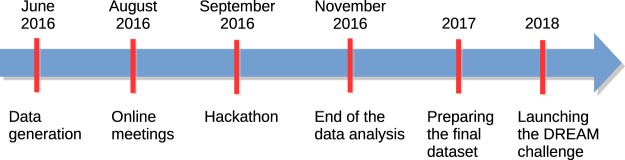
Summary of the timeline of the DREAM of Malaria Hackathon.

A summary of the timeline of the hackathon planning is provided in Figure 5. Planning discussions for organizing the DREAM of Malaria Hackathon commenced in late 2015 based on an NIH grant awarded to the Ferdig Laboratory (University of Notre Dame) on the use of crowdsourcing as a means for identifying mechanisms of artemisinin resistance and potential drug combinations. The hackathon was proposed as an idea to bring together young African scientists to receive training on genomics approaches for predicting drug mechanism of action, resistance, and combinatorial therapies, especially for malaria. One of the staff at IBM Research, who was involved in the original DREAM grant, had collaborations with the University of Notre Dame and initiated the collaboration with H3ABioNet due to the common goals of both IBM and H3ABioNet to build African capacity. The relationship between H3ABioNet and the University of Notre Dame was bolstered by the commitment of the data generators in its grant proposal to the NIH to make the data available prepublication for an open science challenge. This contrasts the common model of data sharing in which data are only shared with a large group after data generators have published the results.

**Figure 6.**
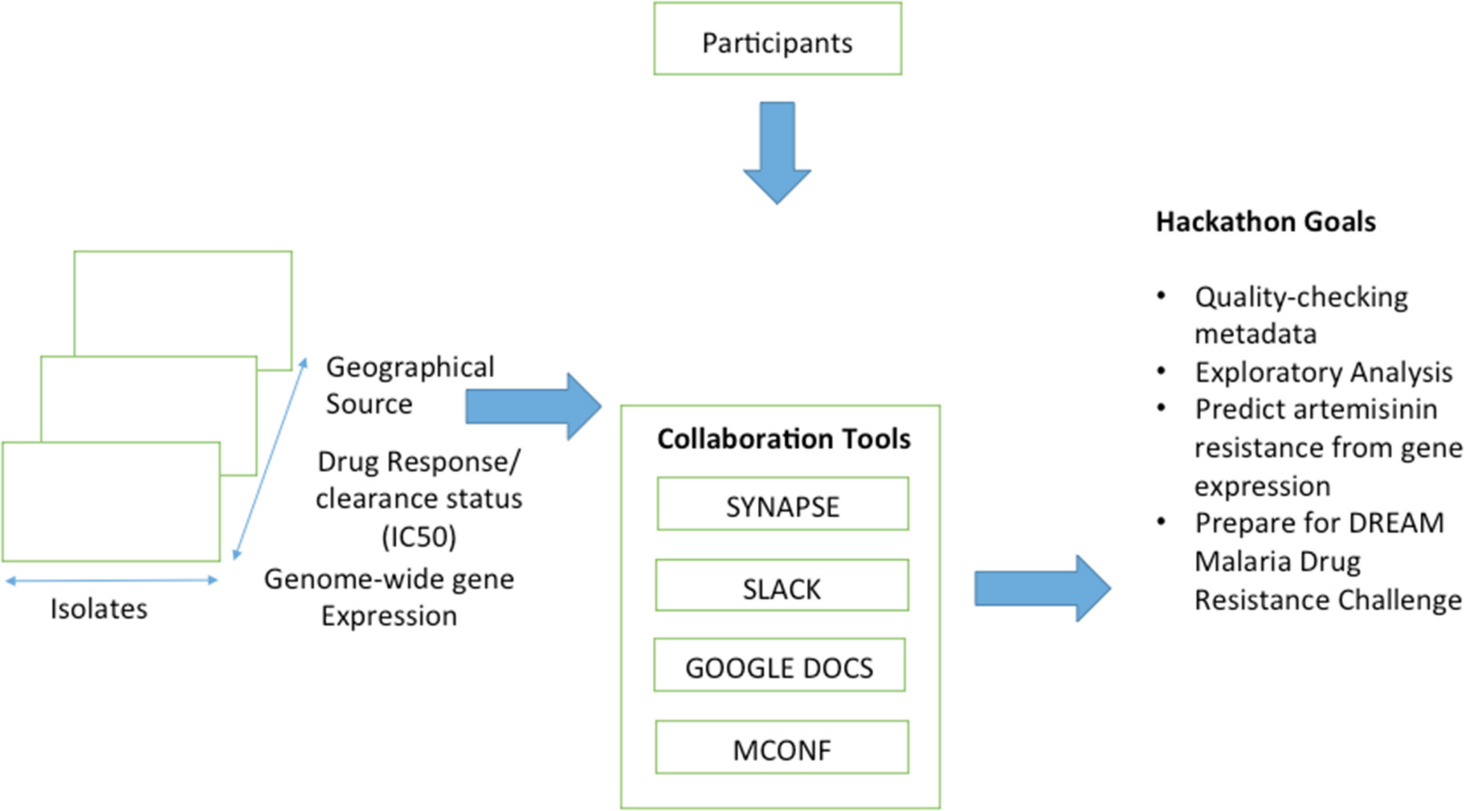
Overview of the main hackathon goals and components.

Hackathon leaders were selected from the three organizations. For H3ABioNet leaders, junior academics were selected who had demonstrated leadership skills and would benefit from the experience of managing the project. H3ABioNet agreed to support the recruitment of participants across its nodes in several African countries to participate in the hackathon. An official call for applications for hackathon participants was placed online in June 2016 and was open for about 3 wk. Forty-eight applications were reviewed by a committee from H3ABioNet. Online meetings took place before the hackathon to initiate the discussion between the participants and to develop a roadmap along with an initial analysis pipeline. The main advantage of this pre-hackathon phase was that the teams became familiar with the data sets and proposed a consensus direction as a team, which facilitated the work at the beginning of the hackathon. Several collaboration tools were used, including MConf for video conferencing, Slack, Synapse, and Google docs. A summary of the main hackathon goals and components, including communication platforms, is shown in Figure 6.

### Data sets

The University of Notre Dame generated several microarray data sets consisting of whole-genome expression for 20 *P. falciparum* isolates coming from Southeast Asia. The scientific goal of this hackathon was to predict the sensitivity of various *P. falciparum* isolates to dihydroartemisinin (DHA) based on genome-wide gene expression profiles of the isolates obtained at two developmental time-points in the presence of DHA or under control conditions. Two proxies were used to measure per-isolate sensitivity of the drug: the dichotomous fast/slow clearance status of an isolate (slow-clearing isolates display some resistance to the drug, whereas fast-clearing isolates respond to the drug as expected) and its IC_50_ drug concentration (measuring the amount of drug to be administered to halve the parasite load). The data generators also provided metadata on the samples, including the geographical source of the malaria parasites, presence of specific mutations at key genetic loci associated with the emerging artemisinin resistance in Southeast Asia, experiment batch information, etc. All the data were stored on the Synapse platform (Derry et al. 2012) and were accessible only to participants. It is important to note that the hackathon organizers undertook steps to ensure that the data set used in the hackathon remains confidential until the DREAM crowdsourcing challenge is launched. First, the data are not publicly available and access was only provided to hackathon participants. Second, a separate set of data that will be used for blind testing of models in the DREAM challenge was not available at the time of the hackathon and will be inaccessible even to the hackathon participants.

### Hackathon team composition

The hackathon participants came from diverse scientific backgrounds, including modeling and microarray data analysis, and five with no or minimal experience in modeling or computing. Among the former, some participants displayed strong skills in statistics, others in computing. The participants were also at various stages of their careers, including MS and PhD students, postdoctoral researchers, research associates, and university faculty.

Three heterogeneously skilled teams were formed with diverse and complementary areas of expertise, which created an ideal environment for generating robust ideas by combining the experience and contributions of the different members that could not be achieved by one individual member. Moreover, this multidisciplinary environment covering the different skills required to handle the data sped up the analysis process by getting different members working in parallel on different aspects while encouraging continuous daily peer review and rapid learning.

### A day at the hackathon

The first day of the hackathon was dedicated to a detailed presentation of the different aspects of the data set by the data generators as well as presentations and discussions of the initial analysis plans from the different teams. Teams then split into their individual collaboration areas at the IBM Research Africa laboratory in Johannesburg, South Africa, to plan their analysis strategies. During the days that followed, a report-back session was scheduled at the end of the day, during which each of the teams presented their analysis results and observations to the whole group. This generated interesting open group discussions that provided the different teams with constructive feedback from the data generators and all participants. This helped teams to crystallize ideas and refine their roadmaps.

Although the three different teams achieved significant progress toward the data cleaning and preanalysis during the hackathon, further work was required. The team members committed to contributing and investing time to finalize the analysis after the hackathon. Each team presented a final project plan along with a timeline.

### Conclusion

We recommend this hackathon-based format for preanalysis of genomic data to be widely adopted for the preparation of crowdsourced data mining challenges or any data set that would be open to the community to enhance the quality of the scientific research. Soliciting the contributions of a large community of scientists with different backgrounds and getting them working together is ideal to create a structured environment to tackle a scientific problem in a short period of time and to critically prepare a data set and extract the key features. In addition, it provides a potential framework for training young scientists on computational biology and data science. The hackathon environment also enabled participants to gain a deeper understanding of specific topics, implement critical thinking, and learn by working with others. Furthermore, it fostered communication, teamwork, and presentation skills among participants at different levels of their careers. Hackathons can be useful throughout the life cycle of projects, e.g., at the start of projects for skills development and transfer, before data are generated in order to have computational pipelines in place for processing the data, midway through a project to derive a common version of processed data for analyses by a consortium, and toward the end of the project for analyzing and comparing results and sanity checking. Another post-project application of hackathons could be to replicate results from a publication analysis. H3ABioNet is using hackathons as a means to build and subsequently utilize capacity in bioinformatics in Africa, and to provide a dedicated environment for hackathon goals to be realized. Prior to this hackathon, H3ABioNet organized a Cloud Computing hackathon to develop and containerize reproducible bioinformatics workflows for use in analysis of H3Africa data. H3ABioNet and DREAM of Malaria hope to work together long-term to organize hackathons that address different segments in the research cycle.

## The DREAM of Malaria Hackathon participants

Taoufik Bensellak,^12^ Anita Ghansah,^13^,^22^ Kais Ghedira,^14^,^22^ Ashley Gritzman,^15^ Itunuoluwa Isewon,^16^ Ali Kishk,^17^,^22^ Ahmed Moussa,^18^ Cheikh Loucoubar,^19^ Patrick Musicha,^20^,^22^ Meenal Pore,^21^ David Moinina Sengeh,^21^ Darlington Shingirirai Mapiye,^15^ Pavan Kumar Rallabandi,^15^ and Melvin Varughese,^15^

## Competing interest statement

G.H.S., D.S.M., D.M.S., P.K.R., M.P., A.G., and M.V. were employees of IBM Research Africa at the time this manuscript was submitted.

## Supplementary Material

Supplemental Material

## Footnotes

12 System and Data Engineering Team Abdelmalek Essaadi University, ENSA, 90000 Tangier, Morocco and University of Liverpool, Liverpool, Liverpool L69 7ZB, UK

13 Noguchi Memorial Institute for Medical Research College of Health Sciences, University of Ghana, LG 581 Legon, Ghana

14 Laboratory of Bioinformatics, Biomathematics and Biostatistics (LR16IPT09), Institut Pasteur of Tunis, University of Tunis El Manar, 1002 Tunis, Tunisia

15 IBM Research Africa, 2001 Johannesburg, South Africa

16 Department of Computer & Information Sciences & Nigeria Covenant University Bioinformatics Research (CUBRe) Covenant University, 112233 Ota, Ogun State, Nigeria

17 Faculty of Agriculture, Zagazig University, 44519 Sharkia, Egypt

18 System and Data Engineering Team Abdelmalek Essaadi University, ENSA, 90000 Tangier, Morocco

19 Group of Biostatistics, Bioinformatics and Modeling (G4-BBM), Institut Pasteur de Dakar, 12500 Dakar, Senegal

20 Malawi-Liverpool-Wellcome Trust Clinical Research Programme, Queen Elizabeth Central Hospital, Chichiri Blantyre 3, Malawi

21 IBM Research Africa, Karen 00200 Nairobi, Kenya

22 As members of the H3ABioNet Consortium's Research Working Group, and members of the H3Africa Consortium

## References

[GR228460GHOC1] Angelidis P, Berman L, Casas-Perez ML, Celi LA, Dafoulas GE, Dagan A, Escobar B, Lopez DM, Noguez J, Osorio-Valencia JS, 2016 The hackathon model to spur innovation around global mHealth. J Med Eng Technol 40: 392–399. 27538360 10.1080/03091902.2016.1213903 PMC5681847

[GR228460GHOC2] Derry JMJ, Mangravite LM, Suver C, Furia MD, Henderson D, Schildwachter X, Bot B, Izant J, Sieberts SK, Kellen MR, 2012 Developing predictive molecular maps of human disease through community-based modeling. Nat Genet 44: 127–130. 22281773 10.1038/ng.1089 PMC3643818

[GR228460GHOC3] Groen D, Calderhead B. 2015 Science hackathons for developing interdisciplinary research and collaborations. eLife 4: e09944. 26202977 10.7554/eLife.09944 PMC4511834

[GR228460GHOC4] H3Africa Consortium, Rotimi C, Abayomi A, Abimiku A, Adabayeri VM, Adebamowo C, Adebiyi E, Ademola AD, Adeyemo A, Adu D, 2014 Research capacity. Enabling the genomic revolution in Africa. Science 344: 1346–1348. 24948725 10.1126/science.1251546 PMC4138491

[GR228460GHOC5] Lapp H, Bala S, Balhoff JP, Bouck A, Goto N, Holder M, Holland R, Holloway A, Katayama T, Lewis PO, 2007 The 2006 NESCent phyloinformatics hackathon: a field report. Evol Bioinform Online 3: 287–296.

[GR228460GHOC6] Mulder NJ, Adebiyi E, Alami R, Benkahla A, Brandful J, Doumbia S, Everett D, Fadlelmola FM, Gaboun F, Gaseitsiwe S, 2016 H3ABioNet, a sustainable pan-African bioinformatics network for human heredity and health in Africa. Genome Res 26: 271–277. 26627985 10.1101/gr.196295.115 PMC4728379

[GR228460GHOC7] Nandi A, Mandernach M. 2016 Hackathons as an informal learning platform. In Proceedings of the 47th ACM Technical Symposium on Computer Science Education (SIGCSE ’16), pp. 346–351, Memphis, TN, USA.

[GR228460GHOC8] Stolovitzky G, Monroe D, Califano A. 2007 Dialogue on reverse-engineering assessment and methods: the DREAM of high-throughput pathway inference. Ann N Y Acad Sci 1115: 1–22. 17925349 10.1196/annals.1407.021

