# Supplementary material for: Hackathons as a means of accelerating scientific discoveries and knowledge transfer
Source: Genome Res. 2018 May;28(5):759–65. doi: 10.1101/gr.228460.117 (PMC5932615; doi:10.1101/gr.228460.117)
Supplement: Supplemental Material [file supp_28_5_759__index.html]

Hackathons as a means of accelerating scientific discoveries and knowledge transfer — Hackathons as a means of accelerating scientific discoveries and knowledge transfer — Supplemental Material 

# Hackathons as a means of accelerating scientific discoveries and knowledge transfer

## Supplemental Material

- Supplemental\_File\_S1.pdf
