# Supplementary material for: Hackathons as a means of accelerating scientific discoveries and knowledge transfer
Source: Genome Res. 2018 May;28(5):759–65. doi: 10.1101/gr.228460.117 (PMC5932615; doi:10.1101/gr.228460.117)
Supplement: Supplemental Material [file supp_gr.228460.117_Supplemental_File_S1.pdf]

Supplemental File S1
Hackathon Survey Questions

Section A - Biology of Plasmodium falciparum

1- What is Plasmodium falciparum?
- a bacteria
- A virus
- A pathogen
- A vector
- A parasite

2- Is P. falciparum a unicellular or pluricellular organism?
3- In which organism(s) does P. falciparum live?
4- Which regions of the world is malaria endemic in?
5- Why is it important to synchronise carefully the different cultures involved in a single transcriptomic study?

Section B - Drug Resistance

1- Cite three chemical compounds having been in common use as antimalarials over the last 10 or 20 years.
2- In which regions of the world do we find resistance to artemisinin?
3- At which stage is P. falciparum susceptible to common antimalarial drugs?
4- What is an IC50 measurement?
5- Briefly describe two mechanisms of resistance to ART in P. falciparum.

Section C - Microarrays

1- What biological product are you measuring with gene expression?
2- What is physically measured on microarrays?
3- Why does one have to normalize microarray experiments?
4- What is a biological replicate?
5- What is a technical replicate?
6- Of the two types of replicates, which one is the most relevant to tackle natural variation in the data?

Section D - Modeling

1- What is a classifier?
2- Name three methods of automatic classification.
3- What is the name of the process consisting in fitting a curve (model) to a set of points?
4- In how many subgroups would you split your dataset before running some classification algorithm? Name them.
5- What is the difference between supervised and unsupervised classification?
6- Consider a classifier. What measures would you calculate to assess its performance? Explain what they mean or how you calculate them.
7- What is overfitting?
